# Supplementary material for: Aryl Hydrocarbon Receptor (AhR)-Mediated Signaling in iPSC-Derived Human Motor Neurons
Source: Pharmaceuticals (Basel). 2022 Jul 4;15(7):828. doi: 10.3390/ph15070828 (PMC9321538; doi:10.3390/ph15070828)
Supplement: Supplementary file 1 [file pharmaceuticals-15-00828-s001.zip › pharmaceuticals-1770642-supplementary.pdf]

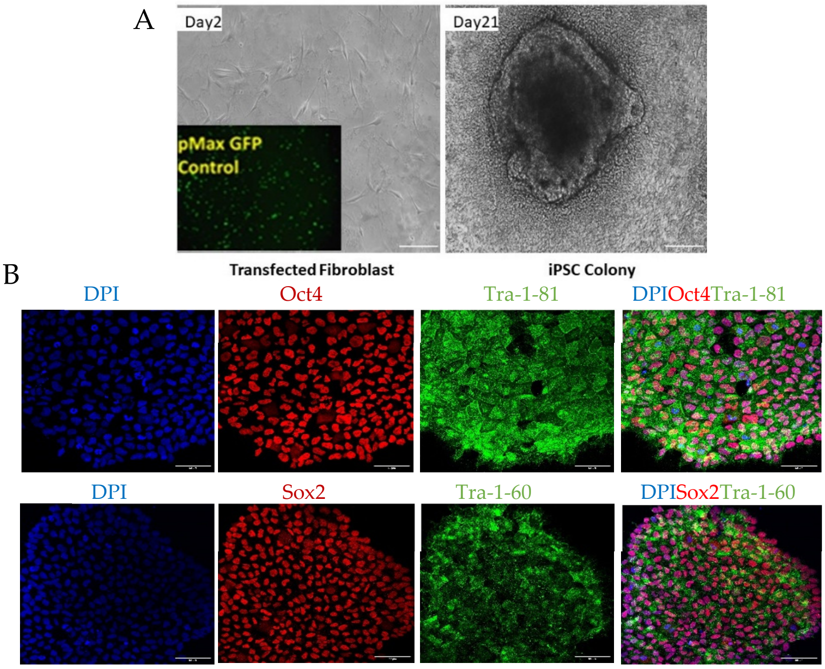

Figure S1: (A) Phase contrast imaging of fibroblasts and the control transfection efficiently with GFP plasmid (in left bottom square) within 24Hrs post transfection and appearance of the hiPSCs colonies after the reprogramming. (b) immunostaining images of the reprogrammed hiPSCs colonies, highly expressed colonies were showing expression of Oct 4 (red), TRA-1-60 (Green) and SOX2 (red). Overlay images are shown merged with nuclear staining with DAPI (blue). Colonies were maintained in recommended media E8 for several passages after picking them from day 21 to Day 30

Table S1. Primer sequences for gene expression analysis

	**Primer sequences from the Universal Probe Library (UPL)**		
Gene Symbol	Forward Primer Sequence	Reverse Primer Sequence	UPL probe Number
CYP1A1	CCAGGCTCCAAGAGTCCA	GATCTTGGAGGTGGCTGCT	33
CYP1B1	ACGTACCGGCCACTATCACT	CTCGAGTCTGCACATCAGGA	20
ARNT1	CCCAACTTGGACCCCTACTAC	TCTTAGCAGTAGCCTGGGTAGC	113
ARNT2	GTCAGACTGAAGTGTTCCAGGAC	CAGTCCCCTGGGTTGGAT	44
GAPDH	CTCTGCTCCTCCTGTTCGAC	ACGACCAAATCCGTTGACTC	60
AhRR	CAAAACCCAGAGCAGACACC	CAGTTCCGATTCGCACAGA	77
AHR	CAGAGTCTGGACAAGGAATTGA	TCTGGAGGAATCTGGTCTGG	54
Calretinin	GCT CCA GGA ATA CAC CCA AA	CAG CTC ATG CTC GTC AAT GT	NA
Hoxb9	TAA TCA AAG ACC CGG CTA CG	CTA CGG TCC CTG GTG AGG TA	NA
Olig2	CAG AAG CGC TGA TGG TCA TA	TCG GCA GTT TTG GGT TAT TC	NA
PAX6	GGG CAA TCG GTG GTA GTA AA	CTA GCC AGG TTG CGA AGA AC	NA
Chat	ACTGGGTGTCTGAGTACTGG	TTGGAAGCCATTTTGACTAT	NA
Sox1	AAT CCC CTC TCA GAC GGT G	TTG ATG CAT TTT GGG GGT AT	NA

*NA: Not applicable

Table S2 : List and dilutions of antibodies used for immunofluorescence staining

Antibody	Company	Catalogue	Dilution
mouse anti-Tra1-60	Cell Signalling (,Technology	4746	1:500
mouse anti-Tra1-81	Cell Signalling Technology	4745	1:500
mouse anti-SSEA4	Cell Signalling Technology	4755	1:500
mouse anti-OCT4	Cell Signalling Technology	2750	1:200
rabbit anti-β3-tubulin, Tuj1	Cell Signaling Technology	8572	1:200
rabbit anti-neurofilament	Cell Signaling Technology	8572	1:200
PSANCAM	Abcam	ab69763	1:400
rabbit anti-MAP2	Cell Signaling Technology	4542	1:400
Mouse anti-AhR antibody	Santa Cruz Biotechnology	sc-133088	1:500
donkey anti-mouse Alexa Fluor 488	Life Technology	A-11094	1:500
donkey anti-rabbit 568	Life Technology	A10042	1:500

Table S3: Active and passive electrophysiological properties of hiPSC-derive neurons.

ectrophysiological properties	Cells with a complex current pattern		Cells with an outwardly rectifying current pattern	
V_m [mV]	-53.3 ± 1.9	(n=23)	-56.3 ± 1.4	(n=64)
IR [MΩ]	591.1 ± 91.2	(n=23)	1497.4 ± 80.7	(n=64)
C_m [pF]	14.4 ± 1.3	(n=23)	10.7 ± 0.3	(n=64)
K_A [pA]	320.5 ± 38.7	(n=7)	288.0 ± 23.0	(n=41)
K_{DR} [pA]	371.8 ± 32.5	(n=23)	582.5 ± 19.3	(n=64)
K_{IR} [pA]	37.9 ± 3.2	(n=23)	no currents	
Na^+ [pA]	189.6 ± 80.0	(n=2)	437.7 ± 31.2	(n=56)

V_m, membrane potential; IR, input resistance; C_m, membrane capacitance; K_A, fast activating outwardly rectifying K^+ currents; K_{DR}, delayed outwardly rectifying K^+ currents; K_{IR}, inwardly rectifying K^+ currents; Na^+, voltage-dependent Na^+ channels; n, number of cells
